# Synthesis of Defective Nanographenes Containing Joined Pentagons and Heptagons

**DOI:** 10.1002/advs.202201000

**Published:** 2022-04-26

**Authors:** Yiyang Fei, Junzhi Liu

**Affiliations:** ^1^ Department of Chemistry and State Key Laboratory of Synthetic Chemistry The University of Hong Kong Hong Kong 999077 P. R. China

**Keywords:** defects, nanographenes, nonalternant rings, pentagon–heptagon pairs, polycyclic hydrocarbons

## Abstract

Defective nanographenes containing joined pentagons and heptagons exhibit striking physicochemical properties from both experimental and theoretical perspectives compared with their pure hexagonal counterparts. Thus, the synthesis and characterization of these unique polyarenes with well‐defined defective topologies have attracted increasing attention. Despite extensive research on nonalternant molecules since the last century, most studies focused on the corresponding mutagenic and carcinogenic activities. Recently, researchers have realized that the defective domain induces geometric bending and causes electronic perturbation, thus leading to significant alteration of the photophysical properties. This review discusses the synthesis and characterization of small nonalternant polycyclic hydrocarbons in the early stage and recent developments in embedding pentagon–heptagon (5–7) pairs into large carbon skeletons through in‐solution chemistry.

## Graphical abstract

Defective nanographenes containing edge‐sharing pentagon–heptagon (5–7) pairs exhibit intriguing physicochemical properties when compared to their hexagonal counterparts owning to perturbation on conjugated *π*‐electron systems. This review presents a clear development progress on the synthesis of this kind of defective nanographenes from the last century to up‐to‐date reported examples, which aims to comprehensively understand their structure‐related properties.

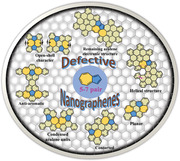

## 1 Introduction

Graphene has a perfect atomic lattice and consists of a planar hexagonal network of all sp^2^‐hybridized carbon atoms. The use of graphene has led to new insights into materials science and technology owing to its extraordinary physicochemical properties.^[^ ^1^, ^2^, ^3^ ^]^ However, structural defects appear to be inevitable in graphene networks. Such structural defects occur because of the second law of thermodynamics, which dictates that disorder occurs in the crystal lattice. These defects also occur during the growth or processing, which includes epitaxial growth on metal surfaces.^[^ ^4^, ^5^, ^6^ ^]^

**Figure 1:**
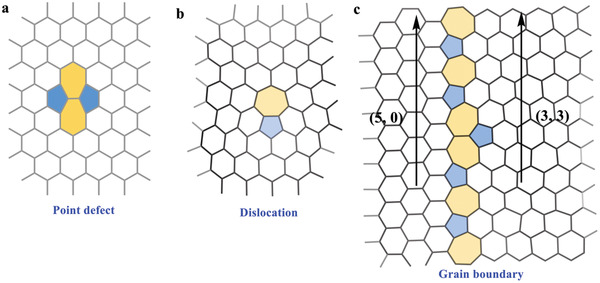
Atomic structures of a) the simplest point defect: Stone–Wales defect, b) dislocation, and c) (5, 0) | (3, 3) class II grain boundary in graphene.^[^ ^9^ ^]^

In general, graphene defects are divided into two categories: 1) intrinsic defects, including point defects (0D), dislocation (1D), and grain boundaries (2D) (**Figure** **1**); and 2) extrinsic defects, such as foreign atoms introduced into the carbon lattice. In recent decades, intrinsic defects in graphene have been extensively studied by experimental observations and/or theoretical calculations, which have shown that defects can lead to electronic perturbations distinct from those of the perfect lattice.^[^ ^7^, ^8^ ^]^ One example of a graphene defect is a class II defect with (5, 0) and (3, 3) matching vectors (Figure 1c),^[^ ^9^ ^]^ in which the linear defect structure contains five‐ and seven‐membered rings, with an extraordinarily large transport gap (*E*_g_) of 1.04 eV, in sharp contrast to the lack of a bandgap in graphene. Unfortunately, the generation of structural defects in graphene by crystal growth,^[^ ^10^ ^]^ irradiation,^[^ ^11^ ^]^ and chemical treatment^[^ ^12^ ^]^ usually occurs randomly and arbitrarily; thus, precise control of the defect location is challenging. From both fundamental and applied perspectives, a thorough understanding of these topological defects will be of great importance. Accordingly, the investigation of well‐defined defects in atomically precise and monodisperse graphene fragments will play a unique role in engineering defects in graphene to elucidate the structure–property relationships.^[^ ^9^, ^13^ ^]^

Among these defects in the fragments of graphene (polycyclic hydrocarbons (PHs), also named nanographenes), pentagon‐ and heptagon‐embedded nanographenes have been extensively investigated. For example, since the discovery of fullerenes in the 1980s,^[^ ^14^ ^]^ nonhexagonal polygons have produced a local Gaussian curvature in the planar carbon network. Generally, pentagons cause a positive (bowl‐shaped) curvature,^[^ ^15^, ^16^, ^17^ ^]^ while heptagons lead to a negative (saddle‐shaped) curvature.^[^ ^18^, ^19^ ^]^ Moreover, the electronic properties in defective nanographenes are different from those in pure hexagonal networks.^[^ ^20^ ^]^ For instance, according to Hückel's rule, pentagons and heptagons can become more stable because they gain additional aromatic stabilization energy when accepting/losing one electron to form a 6*π*‐aromatic cyclopentadienyl anion^[^ ^21^ ^]^/tropylium cation,^[^ ^22^ ^]^ which has also been experimentally supported.

Pentagon–heptagon pairs (5–7 pairs or azulene units), which can be regarded as structural isomer of naphthalene, have often been observed in graphene networks as intrinsic structural defects (Figure 1b). Theoretical calculations describing the 5–7 pairs in the monolayer show that they have relatively low formation energy and can be sufficiently constructed with a stable grain‐boundary structure, leading to pronounced electronic perturbation in the original *π*‐electron system.^[^ ^12^, ^13^ ^]^ However, the abovementioned investigations are severely restricted to characterization within 2D materials and are limited to theoretical studies.^[^ ^23^ ^]^ Consequently, the physicochemical properties induced by these topological defects in graphene remain elusive. Nevertheless, it is reasonable to speculate that such defective graphene molecules could have distinguishable topologies and properties. For instance, the curvature, aromaticity, chemical activities, and photophysical properties of nanographenes could be affected by the introduction of defects. Thus, the atomically precise construction of 5–7 paired defects in nanographenes is necessary for understanding the structure–property relationships. Nevertheless, integrating edge‐sharing pentagons and heptagons into the polyarene framework is a very challenging task because there is a lack of appropriate synthetic methodologies that form two different nonhexagonal rings in the same molecule.^[^ ^24^ ^]^

Pentagon‐ or/and heptagon‐containing nanographenes have been extensively investigated. It is well known that different nonhexagonal polygons integrated into *π* systems exhibit diverse geometries, including bowl‐shaped,^[^ ^15^, ^16^, ^17^ ^]^ saddle‐shaped,^[^ ^18^, ^19^ ^]^ and tub‐shaped,^[^ ^25^ ^]^ and they also exhibit varied electronic configurations. Some reviews on curved nanographenes with five‐ and/or seven‐membered rings have been published recently.^[^ ^17^, ^18^, ^19^ ^]^ Accordingly, this review focuses on defective PHs with edge‐sharing 5–7 pairs, including their synthetic protocols, topological geometries, and physicochemical properties, in terms of recent advances. The main text is categorized into two main parts differentiated by time: pioneering works on the synthesis of nonalternant PHs in the early stage, and their incorporation into a larger carbon skeleton, which has occurred over around the past five years.

## 2 Synthesis of Nonalternant Molecules Containing 5–7 Paired Defects in the Early Stage

Azulene is an isomer of naphthalene and is the smallest nonalternant and nonbenzenoid aromatic compound; it consists of one electron‐rich pentagon and one electron‐deficient heptagon, which endow it with a large dipole moment of approximately 1.08 D.^[^ ^26^ ^]^ Additionally, owing to its unique nonhexagonal topology, azulene exhibits a smaller energy gap and abnormal optical behavior (anti‐Kasha's rule) compared to that of naphthalene as a result of the nonmirror related highest occupied molecular orbital (HOMO) and lowest unoccupied molecular orbital (LUMO).^[^ ^27^, ^28^, ^29^ ^]^ Such behavior leads to its intriguing optoelectronic properties, and azulene has attracted significant attention for use in practical applications since it was first discovered by Piesse in 1863.^[^ ^30^, ^31^ ^]^

Furthermore, a separated azulene unit and aligned azulene clusters have been observed experimentally in 2D graphene and are regarded as structural defects. As mentioned above, the 5–7 pair defect (azulene unit) observed in graphene has a low formation energy and is energetically favorable in thermodynamics over other nonhexagonal defects while exhibiting significantly altered electronic configurations. Incorporation of the analogous 5–7 pair defect into polycyclic systems has triggered increasing research interest owing to its unique defective topology and distinctive conjugated *π*‐electron networks. This section will focus mainly on small nonalternant molecules containing joined pentagonal and heptagonal rings in the early stage and discuss them chronologically.

**Scheme 1:**
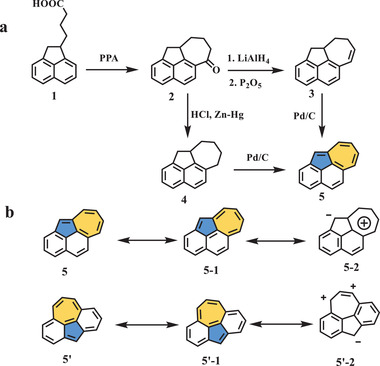
a) Synthesis of compound **5**, and b) resonance structures of **5** and **5′**. PPA: polyphosphoric acid; LiAlH_4_: lithium aluminum hydride; P_2_O_5_: phosphorus pentoxide; HCl: hydrochloric acid.^[^ ^33^ ^]^

Research interests on the 5–7 pair defect in aromatics date back to the early 20th century. Inspired by the unique chemical properties of azulene, Tazuma suggested a resonance hybrid of covalent and betaine forms.^[^ ^32^ ^]^ In 1955, Ward et al. further studied whether azulene retains its original properties after fusion with the phenyl group.^[^ ^33^ ^]^ Accordingly, two structures with different fusion models were selected for preliminary examination: compounds **5** and **5′** (**Scheme** **1**). Based on their resonance forms (Scheme 1b), it was expected that isomer **5** would present typical azulene properties, while **5′** would be a cyclopolyolefin. Structure **5** was successfully obtained from *γ*‐1‐acenaphthenylbutyric acid based on the methodology developed by Sheehan et al. in 1941 (Scheme 1a).^[^ ^34^ ^]^ After several steps, including cyclization of the heptagon, reduction, and dehydrogenation, the final product was isolated as dark red leaf‐shaped particles. The ultraviolet–visible (UV–vis) absorption spectra and basicity measurements demonstrated that hydrocarbon **5** exhibited common azulenic characteristics. Unfortunately, attempts to synthesize compound **5′** failed in this work.

**Scheme 2:**
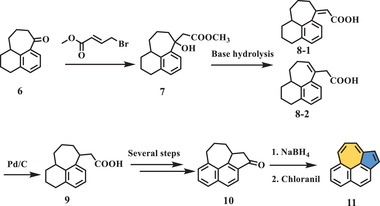
Synthesis of compound **11**. NaBH_4_: sodium borohydride.^[^ ^35^ ^]^

Compound **11**, another isomer of compound **5**, was reported by Osborn et al. in 1957.^[^ ^35^ ^]^ As shown in **Scheme** **2**, starting from 7‐keto‐l,2,3,7,8,9,10,10a‐octahydrocyclohepta[*de*]naphthalene (compound **6**), a large‐scale Reformatsky product (compound **7**) was prepared. Then, dehydration and base hydrolysis were conducted to afford two isomeric unsaturated acids (compounds **8‐1** and **8‐2**). Compound **9** was obtained as a racemic mixture by the reduction of **8‐1** and **8‐2** over Pd/C in acetic acid. Dehydrogenation of the ester of compound **9**, followed by hydrolysis and cyclization, afforded compound **10**, which bore both a heptagon and pentagon ring. Finally, compound **11**, which contained an azulene unit, was synthesized in two successive steps, including reduction of the ketone and aromatization by chloranil. The UV–vis spectrum of compound **11** showed two well‐resolved peaks at 300–350 and 400–440 nm, as well as a broad peak extending from 460 to 650 nm.

**Scheme 3:**
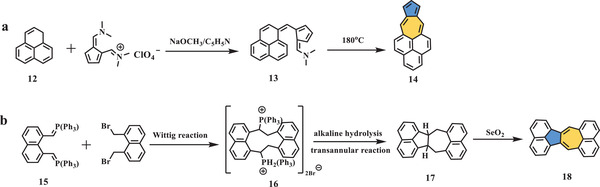
Synthesis of compounds a) **14** and b) **18**. NaOCH_3_: sodium methanolate; C_5_H_5_N: pyridine; SeO_2_: selenium dioxide.^[^ ^36^, ^37^ ^]^

Instead of incorporating an azulene unit with naphthalene, in 1966, Dr. Jutz et al. reported the synthesis of azuleno[5,6,7‐*cd*]phenalene (**Scheme** **3**a),^[^ ^36^ ^]^ in which phenalene was used as the starting reagent. Condensation with *N,N*‐dimethyl‐(6‐dimethylamino fulvenyl methylene) ammonium perchlorate in the presence of methoxide in pyridine afforded a violet phenafulvalene derivative (**13**), which was converted into the final phenalene‐fused azulene (**14**) as green leaflets in moderate yield when heated in quinoline at 180 °C. The structure was confirmed by mass spectrometry and elemental analysis. Further experiments of compound **14** demonstrated azulene‐like properties, for example, intense green fluorescence due to the formation of carbonium ions when it was mixed with acetone in an alcoholic mineral acid solution. Compound **14** exhibited several UV–vis absorption bands with central peaks of 741, 702, 666, 633, 614, 451, 426, and 316 nm.

In 1968, Ruppert et al. used the Wittig reaction to construct various polycyclic compounds, including azulene‐centered compound **18** (Scheme 3b).^[^ ^37^ ^]^ 1,8‐Bis(bromomethyl)‐naphthalene bisphosphonium salt (**16**) was afforded from easily prepared Wittig reagents (**15**); then, hydrolysis under basic conditions accompanied by a transannular reaction gave precursor **17**, which was dehydrogenated by SeO_2_ to produce deep red dinaphthoazulene (**18**). The structure was supported by nuclear magnetic resonance (NMR) and mass spectroscopy, and the photochemical properties were characterized by UV–vis spectroscopy in chloroform, which showed six main peaks at 394, 416, 442, 470, 516, and 550 nm, accompanied by three shoulder peaks at 273, 281, and 300 nm.

**Scheme 4:**
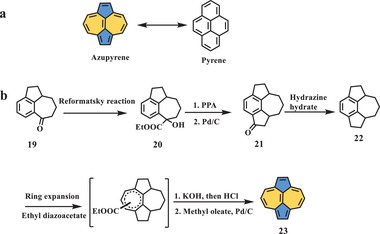
a) Structural isomer of pyrene; b) synthesis of compound **23**. KOH: potassium hydroxide.^[^ ^38^ ^]^

Azupyrene, a structural isomer of pyrene (**Scheme** **4**a), was first synthesized and characterized by Montana et al. in 1968.^[^ ^38^ ^]^ It has a conjugated *π*‐electron system exhibiting nonalternant topology and symmetric geometry (Scheme 4b). The key intermediate **19** was obtained from 1‐indanone after several steps, including the Reformatsky reaction, dehydration, and Friedel–Crafts (F–C) cyclization, and then this intermediate underwent the same steps to afford another ketone (**21**) with an additional five‐membered ring. Treatment **21** with hydrazine hydrate gave key precursor **22**. Subsequently, after a reaction sequence involving a ring‐expansion reaction, hydrolysis, and decarboxylation plus dehydrogenation on Pd/C, azupyrene **23** was obtained as square, bronze platelets with a yield of 2.5%. Its structure was confirmed by NMR and mass analysis. Some investigations, including infrared (IR) spectroscopy and diamagnetic susceptibility were conducted, in which two sharp bands, at 1588 and 1538 cm^−1^, were similar to those in the azulene unit, and the diamagnetic susceptibility was to some extent indicative of the aromaticity of azupyrene. Azupyrene exhibits distinctive electronic properties compared to those of its alternant isomer pyrene owing to its nonhexagonal rings, such as smaller energy gap, more localized charge distribution, and a different conjugation pathway.^[^ ^39^ ^]^ Furthermore, a modified synthetic procedure was developed by Anderson^[^ ^40^, ^41^ ^]^ and Jutz,^[^ ^42^ ^]^ affording the product in moderate yield, and detailed studies involving theoretical calculations^[^ ^43^, ^44^, ^45^ ^]^ and photochemical spectroscopies^[^ ^39^ ^]^ elucidated the influence of the topological structure on the electronic configuration and molecular properties.

**Scheme 5:**
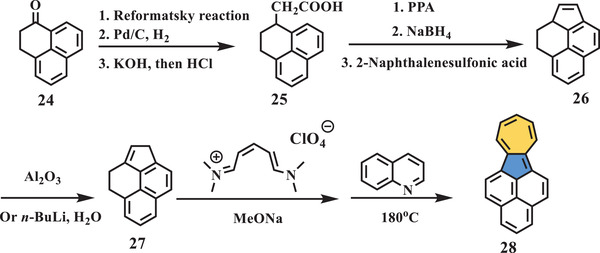
Synthesis of compound **28**. Al_2_O_3_: aluminum oxide; *n*‐BuLi: *n*‐butyllithium.^[^ ^46^ ^]^

Another polycyclic skeleton (compound **28**, **Scheme** **5**), consisting of an azulene unit and a phenalene ring, was reported by Hara in 1975,^[^ ^46^ ^]^ which exhibited electronic properties that were different from those of compound **14**. The UV–vis spectrum showed that the longest wavelength of compound **28** was at 1010 nm, whereas the longest wavelength of compound **14** was located at 739 nm, which indicated a significant difference between these two isomers. Similarly, ketone **24** was used as a key building block to expand the ring skeleton, affording compound **26** in several steps, including the Reformatsky reaction, reduction, hydrolysis, cyclization, and dehydrogenation. Isomeric compound **27** was quantitatively converted from **26** after being passed through an alumina column or from **26** after treatment with *n*‐BuLi followed by quenching with water. Subsequently, a fulvene intermediate was obtained when **27** and dimethyl(5‐dimethylamino‐2,4‐pentadienylidene) ammonium perchlorate were treated with sodium methoxide at room temperature. Cyclization of fulvene at 180 °C afforded azuleno[1,2,3‐*cd*]phenalene (compound **28**) as stable dark red plates with a yield of 62%.

**Scheme 6:**
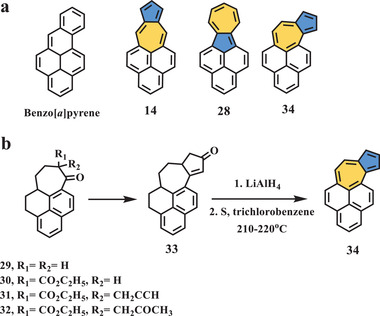
a) Isomeric molecules of benzo[*a*]pyrene; b) synthesis of compound **34**.^[^ ^47^ ^]^

In 1977, Murata et al. reported a third isomer of benzo[*a*]pyrene (compound **34**, **Scheme** **6**b)^[^ ^47^ ^]^ containing azulene and phenalene units. The analogous tetracyclic ketone **29** served as a basic building block for enlarging the ring skeleton, which underwent ethoxycarbonylation, substitution, addition, and intramolecular aldol condensation to afford cyclopentenone **33**. Treatment of ketone **33** with LiAlH_4_ followed by dehydration and aromatization in the presence of sulfur at high temperature resulted in compound **34**, which presented persistent green leaf‐like particles. The diamagnetic susceptibility of **34** was approximately 147 ± 7, which was comparable with that of compound **28**. Moreover, protonation of **34** was conducted in degassed trifluoroacetic acid. As expected for a compound containing an azulene unit, it showed distinct basicity, forming thermodynamically stable cyclohepta[*cd*]phenalenium ions.

**Scheme 7:**
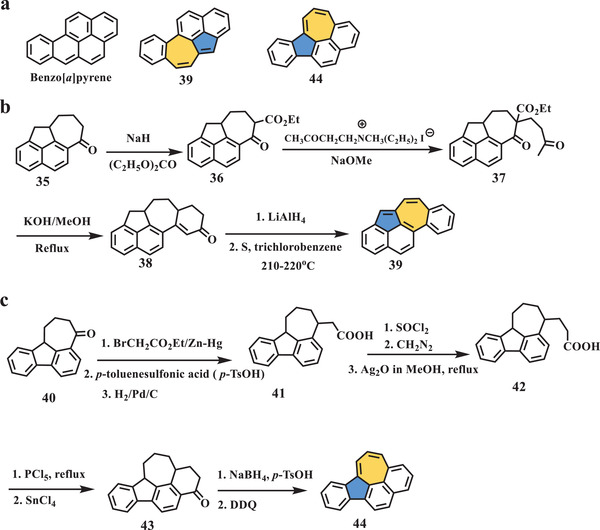
a) Isomeric molecules of benzo[*a*]pyrene; b) synthesis of compound **39**; c) synthesis of compound **44**. NaH: sodium hydride; (C_2_H_5_O)_2_CO: diethyl carbonate; NaOMe: sodium methanolate; SOCl_2_: sulfinyl chloride; CH_2_N_2_: diazomethane; DDQ: 2,3‐dichloro‐5,6‐dicyano‐1,4‐benzoquinone.^[^ ^48^ ^]^

Other isomers of benzo[*a*]pyrene consisting of an azulene unit and benzene rings were reported by Murata in 1979.^[^ ^48^ ^]^ Their nonalternant systems featured charge density distributions that were different from the corresponding alternant isomer (**Scheme** **7**a). The synthesis of compounds **39** and **44** was very similar to that of previously mentioned molecules, including tetracyclic ketones (**35** and **40**, Scheme 7b,c). The precursors containing ketone structures (**38** and **44**) were obtained in a reaction sequence that included several main steps, such as nucleophilic addition, reduction, and cyclization. The treatment of precursors with reducing agents and then aromatization under high temperature afforded the final fully conjugated, red, plate‐like molecules (**39**) and black needle‐like molecules (**44**). Interestingly, compound **39** exhibited a clear blueshift in the absorption spectrum compared to that of compound **5**, which had a smaller conjugated network. However, the absorption spectrum of compound **44** was quite similar to that of compound **11**. Furthermore, compound **39** showed typical basicity and could be reversibly protonated in degassed trifluoroacetic acid, whereas compound **44** was a nonbasic hydrocarbon.

**Scheme 8:**
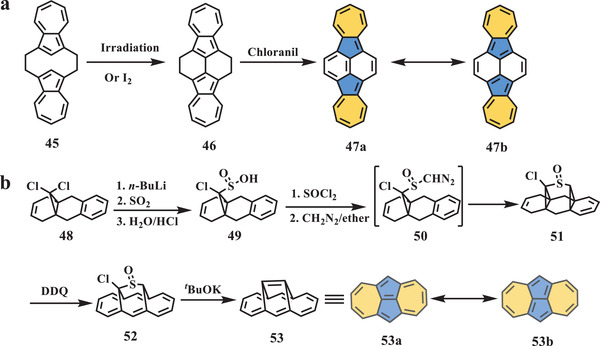
a) Synthesis of compound **47**; b) synthesis of compound **53**. *^t^* BuOK: potassium *tert*‐butoxide.^[^ ^49^, ^52^ ^]^

In 1981, Itô et al. reported a nonalternant isomer (**47**, **Scheme** **8**a) of dibenzopyrene in which two azulene units were connected by a naphthalene core.^[^ ^49^ ^]^ The synthesis started from compound **45**, which can be prepared according to a modified procedure toward pyrene^[^ ^50^ ^]^ and compound **28**,^[^ ^51^ ^]^ followed by irradiation or reaction with I_2_ in dichloromethane (DCM) to afford compound **46** as green needles with yields of 48% and 85%, respectively. Overnight treatment of **46** with chloranil in benzene quantitatively yielded final target **47** as dark violet leaflets. The NMR and electronic spectra of **47**, in which absorption traces consisted of two main absorption peaks at 380 and 580 nm, as well as one shoulder peak around 700 nm, suggested that the [22]annulene resonance structure (**47a**) served as a major contributor rather than the 2,2′‐biazulene, which contained two additional double bonds (**47b**).

Another isomer of pyrene (**53**, Scheme 8b) containing a pentalene subunit was synthesized and characterized by Vogel et al. in 1984,^[^ ^52^ ^]^ while azupyrene **23** contained a heptalene core structure. Although the preparation of **53** had already been achieved, attempts to conduct X‐ray crystallographic analysis failed owing to the low quality of the single‐crystal.^[^ ^53^, ^54^ ^]^ Therefore, the authors employed a novel synthetic route starting from the readily accessible compound **48**. Continuous treatment with butyllithium, sulfur dioxide, and concentrated hydrochloric acid yielded sulfinic acid (**49**), which was exposed to SOCl_2_ and CH_2_N_2_ to yield diazo sulfoxide (**50**). Compound **51** can be easily obtained and separated as colorless crystals from the further decomposition of **50** in situ, followed by dehydrogenation in the presence of DDQ to produce *α*‐chloro sulfoxide **52** as orange needles. The final target, **53**, as greenish‐black needles, can be quantitatively converted from **52** through the Ramberg–Bäcklund reaction. The single‐crystal analysis clearly confirmed the nonalternant structure and substantially corroborated the planar molecular skeleton. Furthermore, the bond analysis revealed that [14]annulene with an additional central double bond (**53a**) was the dominant resonance structure, and this conclusion was also supported by other spectral analyses.

**Scheme 9:**
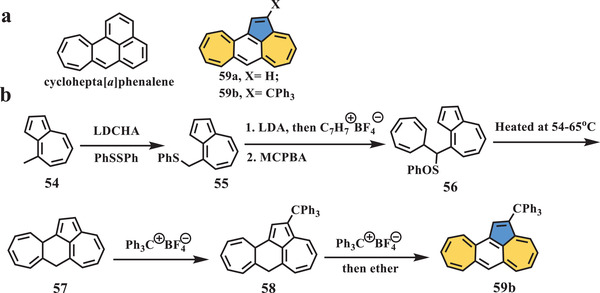
a) Isomeric molecules of cyclohepta[*a*]phenalene; b) synthesis of compound **59b**. LDCHA: lithium dicyclohexylamide; PhSSPh: diphenyl disulfide; LDA: lithium diisopropylamide; MCPBA: *meta*‐chloroperbenzoic acid.^[^ ^55^ ^]^

In 1991, Murata et al. presented a novel nonalternant hydrocarbon^[^ ^55^ ^]^ as an isomer of cyclohepta[*a*]phenalene (**59b**, **Scheme** **9**a) containing azulene and heptafulvene units. The synthesis of compound **59b** involved the stepwise construction of the desired scaffold starting from 4‐methylazulene **54** (Scheme 9b), which was deprotonated by lithium dicyclohexylamide (LDCHA) followed by treatment with diphenyl disulfide to give compound **55**. After introduction of the cycloheptatrienyl group by tropylium tetrafluoroborate and oxidation by *meta*‐chloroperbenzoic acid (MCPBA), a mixture of diastereoisomeric sulfoxides (**56**) was obtained, which was surprisingly converted to the tetracyclic azulene derivative **57** when heated at approximately 55 °C. Owing to the highly reactive site on the free vertex of the pentagon, treatment of **57** with trityl tetrafluoroborate afforded triphenylmethyl‐substituted derivative **58**, and then, sequential treatment with trityl‐tetrafluoroborate and diethyl ether yielded the final fully conjugated compound **59b** as black needles. The UV–vis spectrum exhibited its longest absorption wavelength at 750 nm. From the analysis of the NMR spectrum, it was proposed that the Kekule structure of compound **59b** should sustain a paramagnetic ring current induced by peripheral 16*π* electrons with an external double bond. Cyclic voltammetry (CV) of **59b** showed two oxidation waves at 0.43 and 1.10 V, and one reduction wave at −1.18 V. The first oxidation potential of **59b** was comparable to that of its isomer cyclohepta[*a*]phenalene (*E* ^ox^ = 0.39 V).

In conclusion, because of the lack of effective synthetic protocols for cyclization and incorporation of the 5–7 paired defect into larger carbon skeletons, most of the abovementioned examples applied intramolecular F–C reactions in the presence of strong acids for the cyclization step to form cyclic ketones, and thus, only relatively small PHs were developed during the last century. After F–C acylation, which is normally followed by reduction and dehydration plus aromatization, fully conjugated hydrocarbons can be obtained. Furthermore, analytical techniques during that period are quite limited, which makes it difficult to characterize fully their physicochemical properties and to conduct detailed investigations of the structure‐related differences between alternant and nonalternant topologies. Instead, researchers have focused on the mutagenic and carcinogenic activities of PHs.^[^ ^56^, ^57^ ^]^

## 3 Recent Advances in Synthesis of Defective Nanographenes with 5–7 Pairs

Pioneering works on the chemical synthesis of PHs were conducted by R. Scholl^[^ ^58^, ^59^ ^]^ and E. Clar^[^ ^60^, ^61^, ^62^ ^]^ in the first half of the 20th century and further developed over the century. However, owing to the lack of effective synthetic methods and the limited techniques available for characterization, the synthesis of extended PHs (nanographenes or graphene‐type molecules) with well‐defined sizes and edge structures has remained stagnant for decades, and their corresponding structure–property relations have also been elusive. Up to the beginning of the 21st century, great progress was made in modern organic synthesis and analytic techniques,^[^ ^63^, ^64^ ^]^ especially in metal‐mediated coupling reactions,^[^ ^65^, ^66^, ^67^, ^68^ ^]^ thus allowing the construction of various uniform extended PHs under mild conditions and revealing structure‐related properties such as electronic, optical, and magnetic characteristics. Furthermore, research on large PHs has gained new impetus since the first experimental demonstration of graphene in 2004, as graphene could be used in next‐generation nanoelectronics.^[^ ^69^, ^70^ ^]^ Although much progress has been achieved during the past decade, incorporating 5–7 pair defects into larger carbon skeletons remains challenging because of the lack of a facile synthetic approach for the construction of odd‐membered rings. Additionally, the influence of molecular topology on properties has been gradually demonstrated by theoretical calculations, and its importance has also been increasingly recognized, resulting in revived attention on nonalternant conjugated systems.

Recently, a growing amount of effort has been made to integrate 5–7 pairs into large polyarenes through solution chemistry followed by detailed characterization of their physicochemical properties. Current strategies for the construction of odd‐membered rings, especially for 5–7 pairs in this context, can be classified into several categories including Scholl reactions, F–C cyclization, and metal‐mediated annulation. Moreover, new methods such as photocyclization and *π*‐extension of the azulene unit have recently shown potential. This section will focus on the very recent synthetic advancements toward defective nanographenes containing 5–7 pairs on the basis of a key nonhexagon‐forming step.

### 3.1 Scholl‐Type Cyclodehydrogenation

The Scholl reaction, also called intramolecular oxidative cyclodehydrogenation, has been widely used for the construction of (extended) PHs for over a century since the original report by Scholl et al. in 1910.^[^ ^58^, ^59^ ^]^ Thus far, this reaction has been a versatile tool for the synthesis of various fully conjugated *π*‐electron systems, such as giant nanographenes,^[^ ^71^, ^72^, ^73^ ^]^ graphene nanoribbons,^[^ ^74^, ^75^ ^]^ and twisted nanographenes containing odd‐membered rings.^[^ ^76^ ^]^ Reagents involved in the Scholl reaction normally consist of oxidants and Lewis acid; for example, DDQ/trifluoromethanesulfonic acid (TfOH),^[^ ^77^ ^]^ FeCl_3_,^[^ ^78^ ^]^ Cu(OTf)_2_/AlCl_3_,^[^ ^79^ ^]^ and [bis(trifluoroacetoxy)iodo]benzene (PIFA)/BF_3_·Et_2_O^[^ ^80^ ^]^ have been extensively used in cyclodehydrogenation processes.

However, the mechanism of this C—C bond‐forming reaction remains elusive and controversial^[^ ^81^ ^]^ owing to the unexpected migration of alkyl and/or aryl groups and ring skeleton rearrangement. Very recently, several groups have reported this unusual phenomenon during the Scholl‐type process, which has afforded 5–7 pair defects embedded in nanographene scaffolds, thus allowing for detailed investigation of the unique electronic properties of nonalternant topological structures.

#### 3.1.1 Typical Scholl reaction

**Scheme 10:**
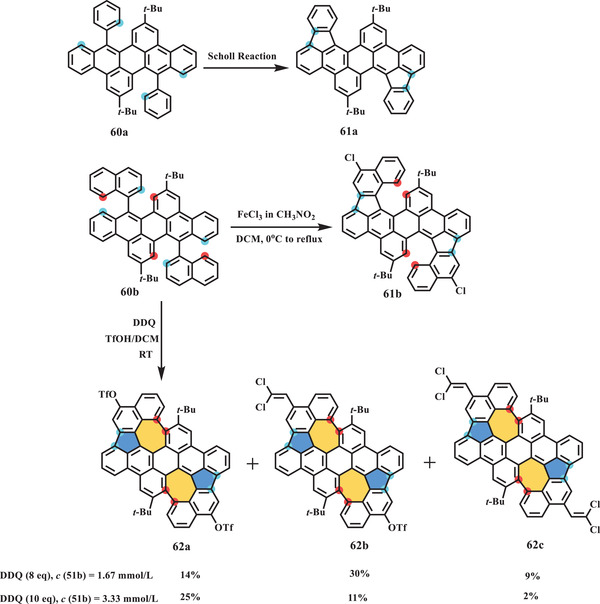
Synthesis of contorted nanographenes **62** with two azulene units.^[^ ^82^, ^83^ ^]^

As one of the most powerful tools for synthesizing nanographenes, Scholl‐type cyclodehydrogenation can form hexagonal rings in most cases, and odd‐membered rings or azulene units embedded in nanographenes have recently been synthesized through this process. In 2019, the Mastalerz group reported the synthesis of contorted nanographenes with two azulene motifs (**Scheme** **10**). On the basis of their previous discovery that Scholl‐type oxidative cyclization of **60a** in the presence of DDQ/TfOH or FeCl_3_/CH_3_NO_2_ exclusively gave the pentagon‐forming product **61a**,^[^ ^82^ ^]^ in this work, the modified precursor **60b** was prepared to conduct the same highly selective Scholl reaction that enabled the generation of azulene‐embedded nanographenes.^[^ ^83^ ^]^ In contrast, treatment of precursor **60b** with FeCl_3_/CH_3_NO_2_, even in a large excess of equivalents, afforded only the pentagon‐containing chlorinated product **61b**. In the presence of DDQ/TfOH, two pentagons accompanied by two heptagons were formed, resulting in three unexpectedly substitutional products **62**, of which the respective yield was dictated by the concentration of DDQ. In particular, bistriflated product **62a** could undergo further postmodification such as a palladium‐mediated cross‐coupling reaction to yield larger aromatics. A high‐quality single crystal of **62c** with two dichlorovinylidene (DCV) groups was obtained by slow vapor diffusion of chloroform/methanol, confirming its negatively curved geometry due to the presence of two heptagons in the scaffold. The bond length analysis demonstrated that the embedded 5–7 pairs in **62c** exhibited longer bonds and more significant alternation patterns than those of pristine azulene units, which indicated less aromaticity. The optical properties of **62** were investigated using UV–vis absorption and emission spectroscopy. The maximum absorption peaks assigned to the S_0_–S_1_ HOMO to LUMO transitions were located in the range of 628–644 nm owing to the gradual extension of the *π* systems, similar to that of azulene. The emission of **62** was shifted to 648–671 nm, close to the near‐IR (NIR) regime, with a quantum efficiency of up to 27%.

**Scheme 11:**
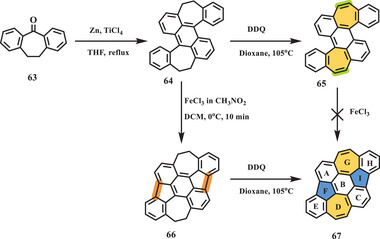
Synthesis of planar nonbenzenoid nanographene **67** with two 5–7 pairs. THF: tetrahydrofuran; TiCl_4_: titanium tetrachloride.^[^ ^84^ ^]^

In 2020, Zhang et al. presented another nonalternant nanographene (**67**) containing two 5–7 pairs through an FeCl_3_‐mediated Scholl reaction (**Scheme** **11**).^[^ ^84^ ^]^ The final compound **67** could be prepared on the gram scale because the synthetic procedure was concise, and purification did not require a column. Starting from dibenzosuberone **63**, TiCl_4_‐promoted dimerization was conducted to afford compound **64**. Interestingly, the reaction sequence was quite important for the transformation of **64** to nanographene **67**, in which Scholl‐type cyclization and dehydrogenation were proven workable. Treatment of **64** with 12 equiv. of FeCl_3_ yielded **66**, which contains additional pentagons; this molecule can be prepared on the gram scale with a high yield of 98% by a purification procedure involving extraction/precipitation. Subsequently, the dehydrogenation of **66** in the presence of DDQ in dioxane followed by physical vapor transport work‐up afforded **67** as a stable black powder. The single‐crystal molecular structure of **67** unambiguously confirmed its nonbenzenoid structure and planar geometry due to the counterbalancing effect of the pentagons and heptagons. The bond length analysis of two conformers of **67** indicated noticeable bond length alternation in the range of 1.333–1.487 Å (conformer 1) and 1.354–1.458 Å (conformer 2), while pristine azulene had a more average distribution of 1.387–1.427 Å, which was also consistent with the LOL‐*π* (localized orbital indicator function) calculation showing *π*‐delocalization over the E/F/B/I/H rings. The UV–vis absorption spectrum of **67** exhibited two bands at 305 to 345 nm and 610 to 666 nm, which were ascribed to the S_0_–S_2_ and S_0_–S_1_ transitions, respectively. Moreover, this azulene‐containing nanographene **67** showed two emission peaks at 400 and 670 nm corresponding to the S_2_–S_0_ and S_1_–S_0_ transitions, respectively. On the basis of density‐functional theory (DFT) calculations, the incorporated azulene unit in **67** makes a major contribution to this abnormal anti‐Kasha emission. Furthermore, the electronic properties of **67** were investigated by CV and the construction of organic field‐effect transistors, indicating multiple redox abilities and p‐type semiconducting behavior, with a low average charge mobility of 0.049 cm^−2^ V^−1^ s^−1^ due to the disordered packing.

**Scheme 12:**
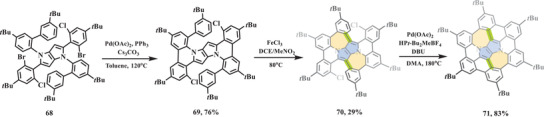
Synthesis of bowl‐shaped nitrogen‐doped nanographene **71** with two 5–7 pairs. PPh_3_: triphenylphosphine; Cs_2_CO_3_: cesium carbonate; Pd(OAc)_2_: palladium diacetate; [HP*t*‐Bu_2_Me][BF4]: di‐tert‐butyl(methyl)phosphonium tetrafluoroborate; DBU: 1,8‐diazabicyclo[5.4.0]undec‐7‐ene.^[^ ^85^ ^]^

In 2021, Gryko et al. reported the synthesis of bowl‐shaped nitrogen‐doped nanographene (**71**) through in‐solution strategy (**Scheme** **12**),^[^ ^85^ ^]^ which was also achieved via on‐surface synthesis.^[^ ^86^ ^]^ In this context, tetraarylpyrrolopyrrole **68** (TAPP **68**) substituted with biphenyls, bearing bromine and chlorine atoms, was obtained from one‐pot multicomponent condensation.^[^ ^87^ ^]^ Subsequently, TAPP **68** was subjected to Heck cyclization under relatively mild condition to selectively give compound **69**. Then heptagon‐forming reaction was conducted through Scholl‐type oxidative cyclodehydrogenation in the presence of FeCl_3_ to yield compound **70** with two remaining C(sp^2^)—Cl bonds. Finally, the target **71** was offered through Pd‐mediated intramolecular cyclization. X‐ray crystallographic analysis of **71** revealed bowl‐shaped geometry with the bowl depth of 2.05 Å, which was deeper than previously reported azacorannulenes (1.70 Å^[^ ^88^ ^]^ and 1.73 Å^[^ ^89^ ^]^). UV–vis spectrum of **71** exhibited complex features with three‐band: the first intense band located between 280 and 350 nm, accompanied by a shoulder peak extending to ≈390 nm; the second‐band was in the range of 390 and 470 nm centered at 399 and 455 nm; as for third weak band, it showed an absorption tail extending to 580 nm from maximum of 500 nm, which can be ascribed to S_0_–S_1_ transition. Furthermore, electronic behavior of **71** was performed by CV, which presented two reversible oxidation waves at *E*_1/2_^ox1^ = −0.09 and 0.30 V (vs. Fc/Fc^+^) and no reduction wave.

#### 3.1.2 Ring‐Skeleton Rearrangements

Very recently, a few examples of ring‐skeleton rearrangements have been reported under Scholl‐type conditions, especially rearrangements under the condition of DDQ/TfOH or DDQ/MsOH (methanesulfonic acid). In the most recent example, presented by Wu et al. in 2021,^[^ ^90^ ^]^ FeCl_3_‐mediated oxidative cyclization, which underwent a radical cation mechanism, led to the formation of a planar dibenzo‐*peri*‐hexacene derivative. Meanwhile, treatment of the same precursor with DDQ/MsOH afforded an unexpected octagon‐containing product with negative curvature through an arenium ion pathway. In addition to the formation of octagons under similar oxidative conditions, nanographenes containing 5–7 pairs can be generated through ring‐skeleton rearrangement.

**Scheme 13:**
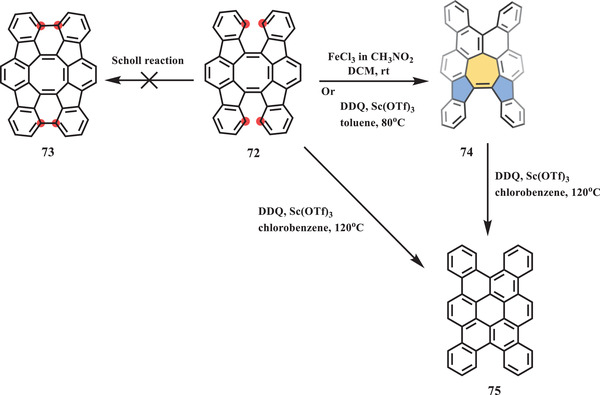
Skeletal rearrangement towards saddle‐shaped nanographene **75** with 5–7–5 core. Sc(OTf)_3_: scandium trifluoromethanesulfonate.^[^ ^91^ ^]^

In 2017, Tobe et al. reported a stepwise skeletal rearrangement from a cyclooctatetraene (COT) derivative to a 5–7–5 core and finally to all hexagonal rings under Scholl‐type conditions.^[^ ^91^ ^]^ Initially, the authors attempted to synthesize a novel [8]circulene analog **73** from COT congener **72** through cyclodehydrogenation (**Scheme** **13**). However, treatment of **72** with either FeCl_3_/CH_3_NO_2_ or DDQ/Sc(OTf)_3_ afforded the unexpected product **74**, which had a 5–7–5 core. Further experiments demonstrated that skeletal rearrangement could occur at higher temperatures in the presence of DDQ/Sc(OTf)_3_, generating all hexagonal frameworks **75** from either pristine COT congener **72** or heptagon‐containing **74** (Scheme 13). DFT calculations indicated a lower energy barrier for skeletal rearrangement than that of the oxidative bond‐forming process. Single‐crystal analysis of **74** showed a saddle‐shaped geometry owing to steric hindrance. Furthermore, the electronic properties of **74** were investigated by CV, which exhibited only two reversible reduction waves at −1.59 and −2.08 V (vs. Fc/Fc^+^), indicating a tendency toward chemical oxidation.

**Scheme 14:**
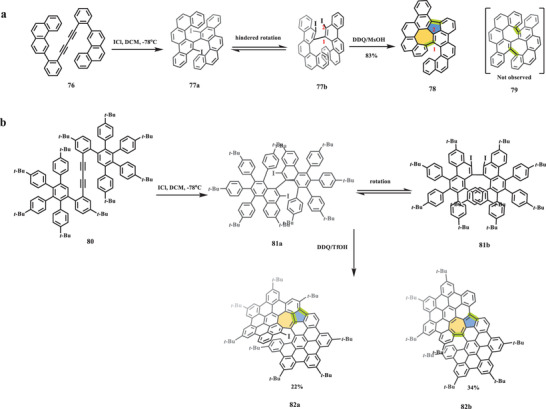
Synthesis of helical nanographenes a) **78** and b) **82** with embedded azulene unit. ICl: iodine monochloride.^[^ ^92^ ^]^

In 2020, a class of unprecedented helical nanographenes containing azulene moiety was reported by Feng and Liu through an unexpected Scholl reaction (**Scheme** **14**).^[^ ^92^ ^]^ Precursor **77** was prepared from diacetylene **76** through selective ICl‐induced cyclization. Treatment of **77** with DDQ/MsOH exclusively generated unexpected helical nanographene **78** containing an azulene unit with a good yield of 83% instead of the projected heptalene‐embedded compound **79** (Scheme 14a), while treatment of **77** with DDQ/TfOH afforded an elusive byproduct. The proposed mechanism of the formation of helical nanographene **78** involves the hindered rotation toward the favored form (**77b**) of **77**, followed by skeletal rearrangement (1,2‐aryl migration) and HI elimination during Scholl‐type oxidative cyclization. To further investigate this interesting result, a larger and flexible precursor **81** was rationally designed and prepared by a reaction sequence that included Diels–Alder cycloaddition and ICl‐mediated benzannulation (Scheme 14b). Treatment of **81** with DDQ/TfOH yielded two main products, **82a** and **82b**, both of which contained azulene units in the core structure. The reason why two different products were formed during the Scholl reaction was the flexibility of precursor **81** compared to that of **77**. In contrast, treatment of analog **81** with DDQ/TfOH but without aryl iodides resulted in a complex and unpurifiable mixture, which indicated that iodine atoms played a key role during the Scholl reaction. Single‐crystal analysis demonstrated that all final compounds (**78** and **82**) exhibited highly helical conformations due to the central heptagon‐containing [6]helicene. Furthermore, the embedded azulene units in these helical nanographenes were found to be highly twisted due to the steric hindrance; for example, compound **82b** has a record‐high twisting angle (16.1°). In combination with bond length analysis and DFT calculations, the inner azulene moiety showed weak aromaticity. The optical and electronic properties of **78** and **82** were investigated by UV–vis spectroscopy and CV, and the calculated optical and electronic energy gaps were consistent with each other. Interestingly, superhelicene **82b** featured six reversible oxidation waves with *E* ^ox^_1/2_ potentials of 0.31, 0.48, 0.87, 1.08, 1.23, and 1.37 V, accompanied by two reversible reduction waves with *E* ^red^_1/2_ at −1.69 and −2.09 V, indicating its amphoteric redox properties.

**Scheme 15:**
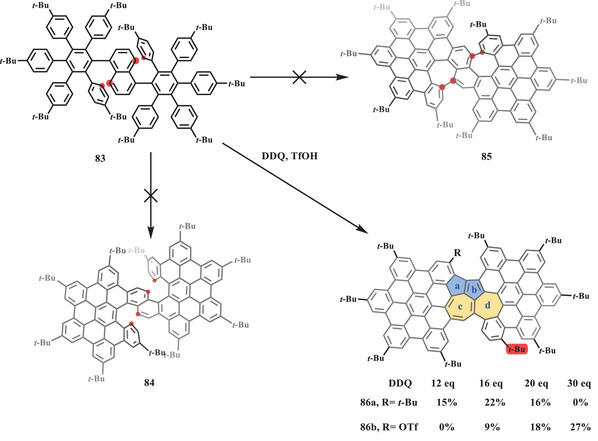
Synthesis of nanographene **86** containing two azulene units.^[^ ^93^ ^]^

Another skeletal rearrangement from naphthalene to azulene was reported by Chi et al. in 2020.^[^ ^93^ ^]^ Their initial idea was the synthesis of the partially fused double [6]helicene **84** and fully fused heptagon‐embedded framework **85** (**Scheme** **15**). Surprisingly, inverse rearrangement from thermodynamically favorable naphthalene to azulene was observed during the Scholl‐type oxidative cyclization instead of the formation of either **84** or **85**, thus affording curved nanographene **86** containing two formal azulene units. Precursor **83** was prepared via a reaction sequence including Sonogashira coupling and a typical Diels–Alder reaction, with a yield of 42% in two steps. Then, after treatment of **83** with DDQ/TfOH at a diluted concentration, two main products were obtained and determined: azulene‐embedded **86a** and its triflyloxylated product **86b**. It should also be noted that the yield of these two products highly depends on the amount of DDQ; for example, when the amount of DDQ was increased from 12 to 30 equivalents, the yields of **86a** and **86b** changed in the ranges of 15% to 0% and 0% to 27%, respectively. This regioselective triflation was also observed by the Mastalerz group.^[^ ^81^ ^]^ X‐ray analysis of a single crystal of **86b** revealed its unique structure with two adjacent azulene units. Furthermore, one *tert*‐butyl group was replaced by a triflate group, while another one was shifted to the bay position through the proposed arenium ion pathway. The bond length analysis and DFT calculations demonstrated that only 5–7–7 pairs (rings b and c) exhibited aromatic properties similar to those of the pristine azulene, which was also supported by the optical spectra including an absorption tail up to 800 nm arising from a forbidden HOMO–LUMO transition and weak photoluminescence in DCM.

### 3.2 Intramolecular F–C Reactions

The F–C reaction, one of the most typical methods of forming C—C bonds, has been widely used for intramolecular cyclization in nanographene chemistry. Commonly, the F–C reaction can be classified into two categories: F–C acylation and alkylation. F–C acylation normally occurs under strong acid conditions, such as the presence of polyphosphonic acid (PPA),^[^ ^94^ ^]^ TfOH,^[^ ^95^ ^]^ and MsOH,^[^ ^96^ ^]^ utilizing carbonyl and carboxylic acids as substrates, in which cyclic ketones are generated. Then, after two consecutive reactions involving nucleophilic addition using Grignard and organolithium reagents and SnCl_2_‐mediated dehydroxy reduction, the final desired nanographenes can be obtained. While F–C alkylation usually starts from aldehyde‐containing substrates, electrophilic addition affords alcohol species for Lewis acid (such as BF_3_·OEt_2_)‐mediated cyclization.^[^ ^97^ ^]^ The obtained cyclized product can be dehydrogenated using DDQ or *p*‐chloranil as the oxidant to give fully conjugated nanographenes. In recent years, the F–C reaction has received increasing attention for the construction of odd‐membered rings such as heptagons.

**Scheme 16:**
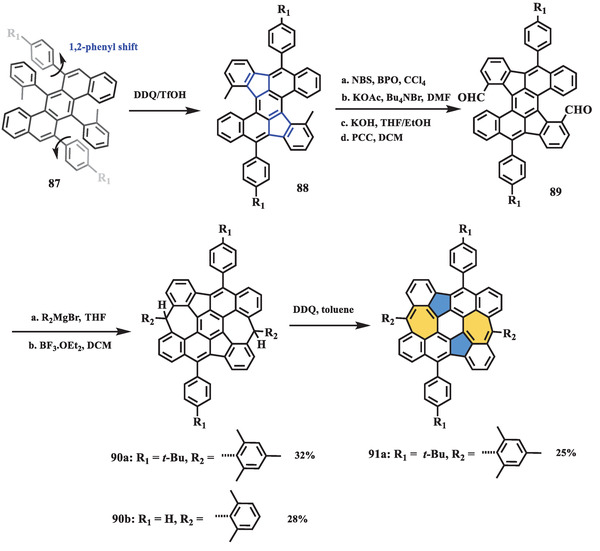
Synthesis of open‐shell nanographene **91** containing two azulene units. NBS: *N*‐bromosuccinimide; BPO: benzoyl peroxide; CCl_4_: carbon tetrachloride; KOAc: potassium acetate; Bu_4_NBr: tetrabutylammonium bromide; DMF: *N*,*N*‐dimethylformamide; PCC: pyridinium chlorochromate; BF_3_·OEt_2_: boron trifluoride diethyl etherate.^[^ ^97^ ^]^

In 2019, Müllen, Fasel, and Feng presented an open‐shell nonalternant nanographene containing two azulene units (**Scheme** **16**).^[^ ^97^ ^]^ In this work, the easily accessible substrate **87** was obtained through several modular steps including the Suzuki coupling, Sonogashira reaction, and ICl‐mediated cyclization, undergoing a 1,2‐phenyl shift in the presence of DDQ/TfOH to afford **88** with two additional pentagons according to their previous finding.^[^ ^98^ ^]^ Then, four consecutive steps involving bromination, esterification, hydrolysis, and oxidation proceeded to achieve transformation from methyl groups to aldehyde groups, affording key precursor **89**. Following routine procedures, including electrophilic addition and BF_3_·OEt_2_‐mediated F–C alkylation and oxidation, the final fully fused compound **91** was obtained as a purple powder, the structure of which was roughly confirmed by high resolution (HR) mass spectrometry. Variable temperature (VT) ^1^H NMR analysis of compound **91** exhibited silencing above −70 °C, while a broad signal appeared when the temperature was reduced below −70 °C, which indicated that triplet species could be easily activated. Accordingly, the magnetic properties of the target were investigated by electron paramagnetic resonance (EPR) and superconducting quantum interference device (SQUID) spectroscopy measurements, and both sets of spectral results indicated singlet biradical character in the ground state, in which the singlet–triplet energy gap (Δ*E*_S–T_) was estimated as −2.15 kcal mol^−1^ through the Bleaney–Bowers equation, close to the value determined via DFT calculation (−1.14 kcal mol^−1^). Furthermore, the optical properties of **91** and its precursor **90** were characterized using UV–vis–NIR spectroscopy. In contrast to compound **90**, which showed a well‐resolved peak, compound **91** exhibited a broad absorption band in the NIR region with a maximum at 936 nm. Accordingly, it had a small energy gap of 1.13 eV. This optical property of **91** is empirically regarded as a characteristic of open‐shell species and can be used for preliminary judgment of whether nanographene has biradical character. The electronic behavior of open‐shell nanographene **91** was studied by CV. Two low oxidation waves at 0.11 and 0.61 V (vs. Ag/AgCl) were observed, indicating that the compound is easily oxidized in an ambient environment.

**Scheme 17:**
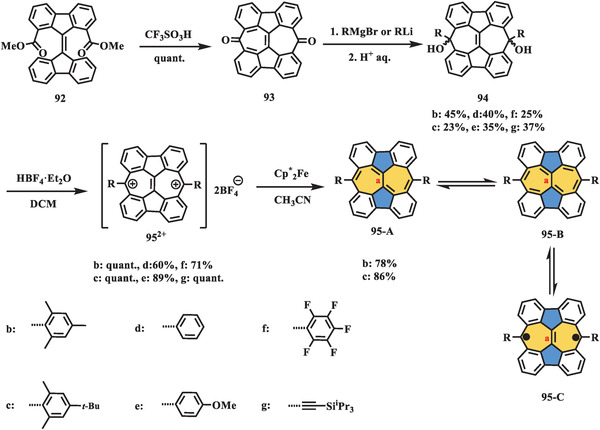
Synthesis of planar open‐shell nanographene **95** with two azulene units. HBF_4_·Et_2_O: tetrafluoroboric acid diethyl ether complex; Cp_2_^*^Fe: decamethylferrocene.^[^ ^95^ ^]^

In the same year, Yasuda et al. reported another nonalternant nanographene (**95**) with open‐shell character in the ground state (**Scheme** **17**).^[^ ^95^ ^]^ Compound **95** exhibited electronic properties that differed from those of its benzenoid isomer, bisanthene. The key F–C acylation precursor **92** was easily obtained in high yield and underwent consecutive reactions, including oxidation, esterification, dichlorination, and Cu‐mediated dimerization, and it could be quantitatively cyclized in the presence of TfOH, yielding dione **93**, which contained two additional heptagons. Subsequently, the treatment of **93** with Grignard and/or organolithium reagents afforded diols **94**. According to a typical procedure, stannous chloride (SnCl_2_)‐mediated dihydroxylation was conducted but afforded only a complete mixture, probably because of the lower aromaticity of target **95**. In contrast, its dication species **95** ^2+^ showed distinct aromatic aromaticity and could be prepared by treatment with a tetrafluoroboric acid diethyl ether complex in quite good yield, and then it underwent two‐electron reduction by decamethylferrocene, giving the desired neutral product **95**. The single‐crystal structures of **95b** and **95c** ^2+^ illustrated a planar and symmetric geometry of the main core due to the surrounding annulated rings. The heptagons in **95b** exhibited more significant bond length alternation than those in **95c** ^2+^, demonstrating the antiaromatic character of the neutral compound **95**. In addition, the length of the central bond ***a*** in **95**b was 1.373(2) Å, which was shorter than the length of a typical C (sp^2^)—C (sp^2^) bond (1.467 Å), indicating that the biradical resonance form (**95‐C**) made a dominant contribution. This result was also supported by DFT calculations and revealed the biradical character (*y*_0_ = 0.72). More experimental evidence was provided by SQUID, EPR, and VT‐^1^H NMR measurements, in which the Δ*E*_S–T_ of **95** was found to be −2.15 kcal mol^−1^ by Bleaney–Bowers fitting of the observed SQUID spectrum, indicating thermal activation to triplet species at low temperature. Electronic and optical behaviors were detected by CV and UV–vis measurements, showing a narrowed energy gap (1.22 eV) and a largely redshifted NIR absorption band at 934 nm, which were related to its benzenoid isomer, bisanthene.

**Scheme 18:**
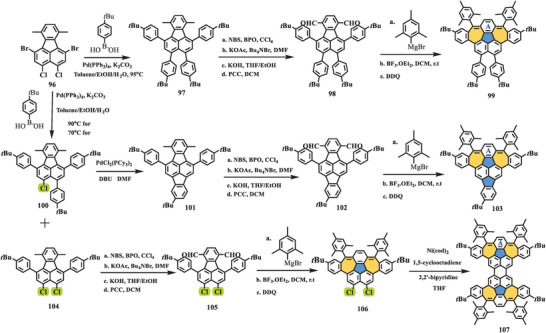
Synthesis of defective nanographenes **99**, **103**, and **107** containing 7–5–7‐membered rings. Pd(PPh_3_)_4_: tetrakis(triphenylphosphine)palladium; K_2_CO_3_: potassium carbonate; PdCl_2_(PCy_3_)_2_: bis(tricyclohexylphosphine)palladium(II) dichloride; DMF: *N,N*‐dimethylformamide; Ni(cod)_2_: bis(1,5‐cyclooctadiene)nickel(0).^[^ ^99^ ^]^

Very recently, Liu et al. presented a novel class of defective nanographenes containing 7–5–7‐membered rings (**Scheme** **18**),^[^ ^99^ ^]^ in which these three nonalternant molecules exhibited antiaromatic characteristics in contrast to the aromatic benzenoid analog dibenzocorannulene. The synthesis started from the key building block **96**, which afforded three different substituted Suzuki products (**97**, **100**, and **104**) depending on the reaction temperature. After a Pd‐catalyzed Heck reaction of **100**, the obtained **101** with additional pentagon accompanied by **97** and **104** followed a typical benzylic oxidation process to give dialdehydes **98**, **102**, and **105**. Subsequently, the treatment of the dialdehydes with mesitylmagnesium bromide followed by F–C alkylation and dehydrogenation yielded heptagon‐fused conjugated nanographenes **99**, **103**, and **106** with yield of more than 90% in three steps. Finally, the dimerization of **106** via the Yamamoto reaction yielded compound **107**, which contained two pairs of 7–5–7‐membered rings, as a dark red solid. Single‐crystal analysis revealed that all three nanographenes possessed 7–5–7 moieties. Compound **99** adopted a nearly planar structure, while compound **103**, which contained an additional five‐membered ring, displayed a slightly saddle‐shaped geometry with torsion angles of 21.6° and 20.4° due to steric hindrance from the armchair region. Interestingly, compound **107**, which can be seen as a head‐to‐head fusion of compound **99**, exhibited a different solid‐state structure, featuring two saddle‐shaped geometries. Furthermore, bond length analysis demonstrated that ring A in nanographenes **99** and **107** showed pronounced alteration of the *p*‐quinodimethane skeleton, while this phenomenon was not observed in **103**, indicating the contribution of the open‐shell form. DFT calculations revealed a small biradical character (*y*_0_ = 0.29); however, VT‐NMR, EPR, and SQUID measurements suggested a closed‐shell character of **103** in the ground state. Theoretical studies further supported the local aromaticity of 7–5–7 embedded nanographenes **99**, **103**, and **107**. The optical properties were investigated by UV–vis and time‐resolved absorption (TA) spectroscopy. Compounds **99** and **103** exhibited similar absorption patterns, in which both showed two main peaks (310 and 520 nm for **99**; 310 and 569 nm for **103**) and two shoulder peaks (455 and 490 nm for **99**; 492 and 530 nm for **103**), respectively; while a broad shoulder peak located at 582 nm was observed in compound **107** compared to its “subunit **99**”. In contrast, the TA spectra of all three compounds were explicitly different from each other, suggesting their distinctive singlet excited states owing to different molecular topologies.

**Scheme 19:**
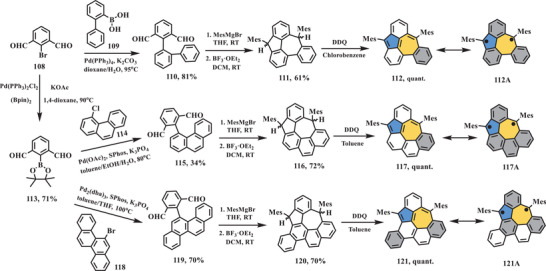
Synthesis of nonalternant PHs **112**, **117**, and **121**. MesMgBr: mesitylmagnesium bromide; Pd(PPh_3_)_2_Cl_2_: bis(triphenylphosphine)palladium(II) chloride; (Bpin)_2_: bis(pinacolato)diboron; SPhos: 2‐dicyclohexylphosphino‐2′,6′‐dimethoxy‐1,1′‐biphenyl; K_3_PO_4_: potassium phosphate tribasic; Pd_2_(dba)_3_: tris(dibenzylideneacetone)dipalladium(0).^[^ ^100^ ^]^

Very recently, three benzo‐extended cyclohepta[*def*]fluorene derivatives (**112**, **117**, and **121** in **Scheme** **19**) exhibiting open‐shell singlet ground state were reported by Feng et al.^[^ ^100^ ^]^ In this work, 5–7 pair was constructed in the latter stage through F—C alkylation followed by dehydrogenation. To this end, key dialdehyde intermediates (**110**, **115**, and **119**) were synthesized by Suzuki coupling in a yield of 81%, 34%, and 70%, respectively. Subsequently, general nucleophilic addition with MesMgBr and followed by intramolecular F–C alkylation of compounds **110**, **115**, and **119** in the presence of BF_3_·Et_2_O afforded dihydro‐precursors **111**, **116**, and **120**, respectively. Final, conjugated compounds **112**, **117**, and **121** were obtained through oxidative dehydrogenation in the presence of DDQ, which were confirmed by HR–mass spectrometry. Open‐shell characters of these three molecules were supported by UV–vis–NIR, VT‐^1^H NMR, and EPR analyses. The UV–vis–NIR absorption spectra of **112**, **117**, and **121** exhibited significantly low‐energy absorption maximum at 1700, 1860, and 1220 nm, respectively, leading to quite narrow optical energy gap of 0.54, 0.52, and 0.69 eV, which are in consistence with DFT calculations. Furthermore, continuous‐wave EPR (cw‐EPR) and VT‐EPR measurements revealed the singlet ground state of nonalternant PHs **112**, **117**, and **121** with low‐lying triplet states and small Δ*E*_S–T_ (−0.04 kcal mol^−1^ for **112**, −0.015 kcal mol^−1^ for **117**, and −0.002 kcal mol^−1^ for **121**), which also supported by VT–^1^H NMR results. However, DFT calculations demonstrated triplet ground states for PHs **112** and **117**, singlet state for PH **121**, which mostly can be ascribed to imperfect energy calculation of corresponding functions.

### 3.3 Metal‐Mediated Annulation

#### 3.3.1 Alkyne‐Based Cyclization

**Scheme 20:**
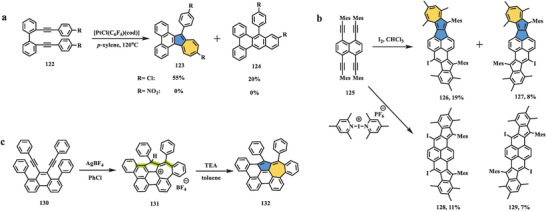
Synthesis of nonalternant nanographenes containing azulene units by alkyne‐based cyclization: a) compound **123**, b) compounds **126** and **127**, and c) compound **132**. AgBF_4_: silver tetrafluoroborates; PhCl: chlorobenzene; TEA: triethylamine.^[^ ^101^, ^105^, ^106^ ^]^

Alkyne‐based benzannulation has long been an efficient strategy for the construction of extended PHs. Recently, defective nanographenes were obtained through unexpected rearrangement, although most of the reported cases were accessible to an all‐benzenoid framework. In 2013, Murakami et al. presented an interesting skeletal rearrangement reaction of 2,2′‐di(arylethynyl)biphenyls toward azulene‐containing nanographene (**Scheme** **20**a).^[^ ^101^ ^]^ In this work, the authors attempted to synthesize a pyrene skeleton using 2,2′‐dialkynylbiphenyl as an alternative substrate via platinum‐catalyzed intramolecular hydroarylation.^[^ ^102^ ^]^ However, two asymmetric nanographenes rather than symmetric pyrene were separated by column. Minor product **124** was determined by NMR and HR mass spectroscopy. The structure of main product **123** was confirmed by single‐crystal X‐ray diffraction, which showed the azulene unit in the skeleton where heptagon was formed by the expansion of one phenyl ring of **122**. The proposed mechanism explained that the reaction undergoes a cationic process. Once vinylic cations are formed, the 4‐chlorophenyl ring attacks two sites intramolecularly, which accordingly results in two different products. Moreover, the cation‐mediated process could be supported by changing the substitution groups to nitro groups with strong electron‐withdrawing ability, in which no reactions were observed and the starting reagents remained intact.

In 2016, Tobe et al. reported another unexpected alkyne cyclization that proceeds in the presence of I_2_ via the electrophile‐induced transformation reported by Wang^[^ ^103^ ^]^ and Wu,^[^ ^104^ ^]^ which formed azulene‐containing nanographenes **126** and **127** (Scheme 20b).^[^ ^105^ ^]^ The treatment of **125** with bis(2,4,6‐trimethylpyridine)iodine(I) hexafluorophosphate afforded the projected product (**128**) and its diastereomer (**129**), which had an indene substructure; these products were expected to have small biradical character according to DFT calculations. Single‐crystal analysis of **126** revealed that azulene and indene were fused on two sides of the molecular skeleton. UV–vis spectra of compounds **126** and **127** exhibited the longest absorption at 760 and 806 nm, respectively; a CV analysis in DCM (vs. Fc/Fc^+^) showed two reversible oxidation waves at +0.22 and +0.68 V for **126** and +0.15 and +0.59 V for **127**, respectively. In combination with the experimental results and theoretical calculations, the reaction mechanism of the generation of compounds **126** and **127** underwent two different models of cyclization at each reactive site, in which an azulene unit was formed by a radical process, while the formation of an indene moiety was proposed to follow the cationic pathway.

In 2018, Yasuda and Konishi reported a tandem oxidative transannulation toward azulene‐embedded nanographene **132** (Scheme 20c).^[^ ^106^ ^]^ In this work, phenanthrene **130** was prepared according to a previously reported method,^[^ ^107^ ^]^ and then treatment with AgBF_4_ selectively afforded compound **132** with an azulene moiety via a two‐electron oxidation process. X‐ray analysis of intermediate **131** revealed the presence of azulenium, indicating that three C—C bonds (highlighted by the green color) between the phenyl and ethynyl groups were formed in one step. Accordingly, a plausible mechanism based on the theoretical calculations was proposed that weak coordination between silver (I) and two acetylene units is believed to promote electron transfer from acetylene to silver (I) ions. Subsequently, tandem cyclizations involving electrophilic and radical processes afforded azulene‐embedded nanographene **132**. The single‐crystal molecular structure of **132** demonstrated a slightly warped saddle‐like geometry, and bond length analysis of the azulene substructure in the molecular skeleton indicated a greater contribution of the pentafulvene unit. The optoelectronic spectrum of **132** featured a redshifted and broad absorption band in contrast to that of its all‐benzenoid isomer. The redox properties of **132** were measured by CV, and the voltammograms exhibited one reversible oxidation and reduction wave centered at *E*_1_^ox^ = +0.47 and *E*_1_^red^ = −1.63 V (vs. Fc/Fc^+^), indicating highly amphoteric redox properties.

**Scheme 21:**
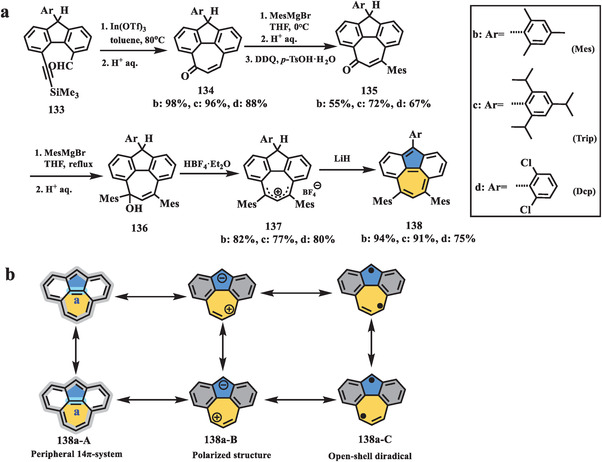
Synthesis of nonalternant isomer of pyrene **138**. In(OTf)_3_: indium(III) triflate; LiH: lithium hydride.^[^ ^108^ ^]^

Very recently, an elusive isomer of pyrene, bis‐periazulene (**138**), was successfully synthesized by Yasuda et al. (**Scheme** **21**a).^[^ ^108^ ^]^ The first attempt toward bis‐periazulene can be dated back to 1955 by Reid,^[^ ^33^ ^]^ and subsequent efforts were dedicated to this target. However, none of them were successful when conducting deprotonation of **137** ^+^·ClO_4_^−^ or two‐electron oxidation of 2Li^+^·**138** ^2−^ owing to the intrinsic instability of bis‐periazulene.^[^ ^109^, ^110^ ^]^ Accordingly, three active sites on **138** were kinetically protected by using bulky aromatic groups to improve their stability in Yasuda's work. From Scheme 20a, compound **134** containing the main skeleton can be prepared from compound **133** through In(III)‐mediated heptagon‐forming cyclization in high yield. Treatment of enone **134** with mesitylmagnesium bromide gave a 1,4‐addition product, which followed by oxidation in the presence of DDQ and *p*‐TsOH·H_2_O afforded compound **135**. Further nucleophilic addition with mesitylmagnesium bromide on carbonyl of **135** yielded alcohol **136**, which was treated by HBF_4_·Et_2_O to produce the key precursor **137**. A suitable base was essential for the final deprotonation process. An over‐reduced radical anion **138c** ^•−^ was obtained in the presence of sodium hydride, while the expected target **138** could be formed when using lithium hydride with a high yield. From the resonance analysis in Scheme 21b, three distinctive electronic structures of **138** can be drawn, including the peripheral 14*π* system (**138a‐A**), charge‐separated polarized structure (**138a‐B**), and open‐shell diradical form (**138a‐C**), which were also supported by experimental and theoretical results. From bond length analysis, the bond length of ***a*** in **138c** between pentagon and heptagon is 1.391 (2) Å, which is significantly shorter than that of pristine azulene (1.489 Å), indicating its double bond character. Combing the harmonic oscillator model of aromaticity, the quinoidal form **138a‐A** was the primary resonance structure compared with **138a‐B** and **138a‐C**. Subsequently, nucleus‐independent chemical shift (NICS (1)), anisotropy of the induced current‐density (ACID), and electrostatic potential (ESP) calculations, along with the experimental results, revealed that the polarized structure (**138a‐B**) cannot be overlooked. Furthermore, SQUID and EPR measurements confirmed its singlet open‐shell character (**138a‐C**), contrary to the previously predicted triplet state of **138**. The UV–vis–NIR spectra of **138** exhibited the longest weak absorption extended to 2000 nm, which is ascribed to HOMO to LUMO transition. Interestingly, the substituent groups showed influence on the Δ*E*_S–T_ of **138**. The Δ*E*_S–T_ of **138d** with the Dcp group was evaluated as −4.6 kcal mol^−1^, five times larger than that of the value of **138c** with Mes group (−0.9 kcal mol^−1^), which may be affected by the electron‐withdrawing property of the Dcp group.

#### 3.3.2 Olefin Metathesis toward Cyclization

**Scheme 22:**
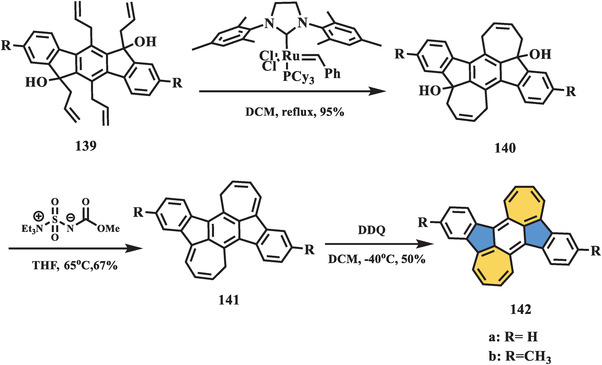
Synthesis of anti‐aromatic molecule **142** containing two azulene units by olefin.^[^ ^111^ ^]^

In 2018, Aso et al. reported the synthesis of fully conjugated PHs containing two azulene moieties, which can also be regarded as a combination of benzo[1,2:4,5]di[7]annulene (BDA) and indenofluorene (IF) subunits in a conjugated framework (**Scheme** **22**).^[^ ^111^ ^]^ In this synthetic process, tetra‐allylated compound **139** was prepared in a reaction sequence that included Suzuki coupling and nucleophilic addition by Grignard reagent. Olefin metathesis mediated by a second generation Grubbs’ catalyst was applied as a key heptagon‐forming step to give pivot intermediate **140** in 95% yield, which was followed by dehydration and oxidation in the presence of Burgess reagent and DDQ, respectively, affording the nonalternant compound **142**. The moderate singlet biradical character (*y*_0_ = 0.49) of **142** arising from the *p*‐quinodimethane core in the IF subunit was predicted by DFT calculations. Furthermore, a large Δ*E*_S–T_ value of 92.5 kJ mol^−1^ was theoretically estimated, indicating the exclusive singlet nature of compound **142**, which coincided with sharp ^1^H NMR peaks. The electronic and optical behaviors of **142** were investigated by CV and UV–vis–NIR spectra, accompanied by theoretical calculations. Both BDA and IF subunits contributed to the electronic character of the fully fused system **142**, but the contribution of BDA was dominant, resulting in a high‐lying HOMO and small energy gap. In addition, intermolecular electronic interactions were observed in the thin‐film UV–vis–NIR absorption spectrum, which was suitable for carrier‐transporting devices. X‐ray single‐crystal analysis of **142** revealed a nearly planar core structure. The bond length analysis and theoretical investigation again demonstrated a dominant contribution of the BDA unit to the electronic character of **142**.

#### 3.3.3 Pd‐Promoted Annulation

**Scheme 23:**
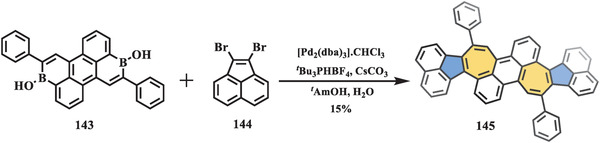
Synthesis of nonalternant nanographene **145** containing two azulene units. [Pd_2_(dba)_3_].CHCl_3_: tris(dibenzylideneacetone)dipalladium‐chloroform adduct; *^t^* Bu_3_PHBF_4_: tri‐*tert*‐butylphosphine tetrafluoroborate; *^t^* AmOH: *tert*‐amyl alcohol.^[^ ^112^ ^]^

Recently, Würthner et al. presented a novel negatively curved polyarene with two pairs of fused pentagons and heptagons (**Scheme** **23**).^[^ ^112^ ^]^ Following their previously developed synthetic strategy toward heptagon‐forming PHs,^[^ ^113^ ^]^ twofold Pd‐mediated [5+2] annulation of 3,9‐diboraperylene (**143**) and 1,2‐dibromoacenaphthylene (**144**) gave a nonalternant molecule (**145**) containing two azulene moieties in a yield of 15%. The electronic properties of **145** were investigated first by CV and square wave voltammetry. Interestingly, the CV traces of **145** featured two low‐lying reversible oxidation waves at 0.00 and 0.20 V (vs. Fc^+/0^), indicating electron‐donating ability. Accordingly, stepwise oxidation was conducted by using nitrosonium hexafluoroantimonate (NO•SbF_6_) to afford radical cation **145** ^•+^ and dication **145** ^2+^, which could be converted back to the neutral compound **145** by treatment with triethylamine. X‐ray crystallography and DFT calculations of **145**, **145** ^•+^, and **145** ^2+^ revealed reorganization of geometries and distinctive electronic configurations. In the solid‐state, the neutral compound **145** and radical cation **145** ^+^ showed a twisted saddle‐shaped structure with cisoid conformations, while **145** ^2+^ adopted a more planar transoid conformation due to the electrostatic interactions between the two counterions. Moreover, the electronic configuration changed upon stepwise oxidation of the curved nanographene with two pairs of fused pentagons and heptagons, which exhibited alternating ring currents.

### 3.4 Photocyclization Process

**Scheme 24:**
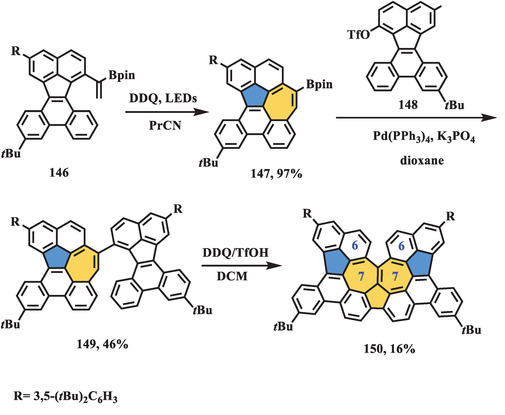
Synthesis of helical nanographene **150** containing azulene cluster. PrCN: butyronitrile.^[^ ^114^ ^]^

Photoinduced cyclization has been widely used to generate phenanthrene substructures from stilbene units in the presence of I_2_ and propylene oxide. However, few examples of heptagon‐forming reactions through photocyclization processes have been reported. In 2020, Takasu et al. presented the synthesis of helical pure carbon‐based nanographene **150** containing continuous azulene units (**Scheme** **24**),^[^ ^114^ ^]^ in which the photoinduced oxidative 10*π*‐electrocyclization process served as the key heptagon‐forming step. Subsequently, the key intermediate **147** bearing a Bpin group underwent a two‐step reaction including Suzuki coupling and Scholl reaction, affording nonalternant nanographene **150**. X‐ray crystallography of **150** revealed a planar edge at the bottom side, which exhibited strong intermolecular interactions resulting in self‐association behavior in solution. The cove region consisting of the 6–7–7–6 ring system had a significantly twisted angle of 46.9° compared to the normal value of 38° in the cove edge. Accordingly, chiral separation was conducted, and two enantiomers were obtained, showing mirror‐symmetric circular dichroism (CD) traces. Theoretical calculations determined a high racemization barrier of **150** up to 29.2 kcal mol^−1^, which was consistent with the experimental result (29.6 kcal mol^−1^). Furthermore, bond length analysis of **150** revealed obvious alternation between 1.39 and 1.49 Å, similar to that in pristine azulene. The aromaticity of the fused pentagon and heptagon estimated by the NICS (1) value was weaker than that of the unsubstituted azulene. The UV–vis spectra of compound **150** showed two main bands at 477 and 404 nm, in which the absorption peak at 477 nm can be ascribed to HOMO to LUMO+1 and HOMO‐2 to LUMO transitions, as well as two weak bands at 660 and 600 nm arising from HOMO to LUMO and HOMO‐1 to LUMO transitions, respectively. The CV trace of compound **150** exhibited three oxidation waves (0.47, 0.69, and 1.0 V) and one reduction wave (−1.5 V, vs. Ag/Ag^+^), which was also supported to be stable even after the oxidation and reduction process.

**Scheme 25:**
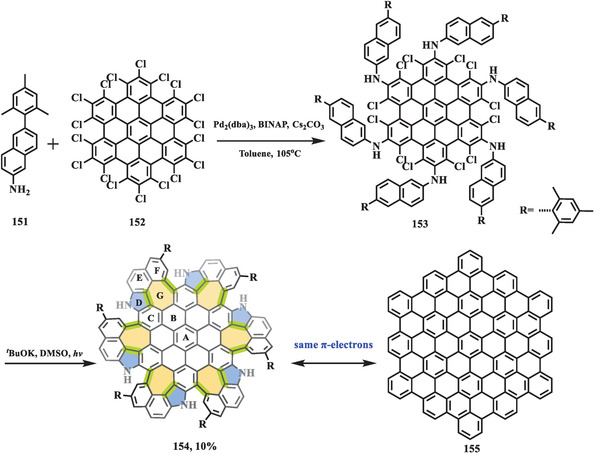
Synthesis of distorted nanographene **154**. BINAP: 1.1′‐binaphthyl‐2.2′‐diphenyl phosphine; DMSO: dimethyl sulfoxide.^[^ ^115^ ^]^

Another photo‐induced heptagon‐forming example was reported by Tan et al. in 2022, in which Monkey‐saddle‐shaped nanographene (**154**) containing up to six joined nitrogen‐doped 5–7 pairs in the carbons skeleton (**Scheme** **25**).^[^ ^115^ ^]^ The key precursor **153** with the remaining 12 chlorines at the bay region was obtained through six‐fold Pd‐mediated C—N coupling between compound **151** and **152**, which was underwent sixfold cascade photo‐induced radical cyclization (PIRC) in the presence of potassium *tert*‐butoxide to afford final wrapped nanographene (**154**). The structure of compound **154** was unequivocally confirmed by ^1^H NMR, HR‐mass, and X‐ray crystallographic analysis, of which revealed Monkey‐saddle‐shaped geometry, as well as S_6_ molecular symmetry. The absorption and emission spectra of **154** exhibited maximum value at 534 and 691 nm, respectively, which was similar to its hexagonal counterpart **155** (blue‐shifted by 5 and 7 nm, respectively), leading to a similar energy gap (1.73 eV for **154**, 1.82 eV for **155**), which can be ascribed to enhanced *π*‐electron delocalization of peripheral rings. Electronic behaviors of **154** and **155** were estimated by CV, in which compound **154** showed a much lower oxidation potential (*E*_1/2_^ox^ = −0.03, 0.13, and 0.31 V for **154**, *E*_1/2_^ox^ = 0.77, 0.80, and 0.95 V for **155**, vs. Fc/Fc^+^) owning to the nitrogen‐doped character.

### 3.5 *π*‐Extension of the Azulene Scaffold

**Scheme 26:**
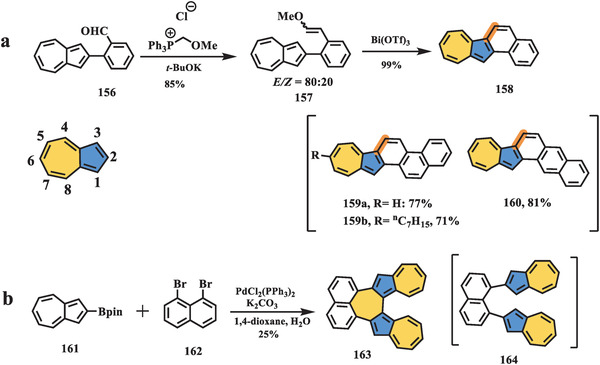
Synthesis of azulene‐fused nanographenes: a) compounds **158**, **159**, and **160**, and b) compound **163**.^[^ ^116^, ^121^ ^]^

Direct extension of the *π*‐system of the parent azulene is a straightforward approach to forming azulene‐embedded polyarenes. However, there are few examples of incorporating formal azulene into conjugated carbon scaffolds owing to the lack of efficient synthetic methods. From the resonance delocalization and distribution of the frontier molecular orbital, the 1 and 3 positions of azulene are the most reactive sites and can easily react with electrophiles, while the 4, 6, and 8 positions (**Scheme** **26**a) in the heptagon exhibit significant electron‐deficiency and accordingly can undergo nucleophilic reactions. Nevertheless, most reactions involving azulene occur on five‐membered rings, while the heptagon in azulene remains elusive.

In 2017, Takai et al. reported the synthesis of three azulene‐fused nanographenes, **158–160**, in four steps (Scheme 26a).^[^ ^116^ ^]^ The key intermediate **156** can be prepared through iridium‐catalyzed borylation and Suzuki coupling, and it is transferred to a mixture of methyl vinyl ester **157** by a typical Wittig reaction, followed by bismuth‐mediated cyclization in a quite high yield of 99% under mild conditions. It is worth noting that this bismuth‐mediated cyclization and aromatization have been powerful tools for the construction of phenacene‐like structures in nanoring and helicene chemistry^[^ ^117^, ^118^, ^119^ ^]^ since their first discovery in 2014.^[^ ^120^ ^]^ Following a similar procedure, extended nanographenes **159** and **160** were obtained successfully. The photophysical properties of azulene‐fused systems **158–160** were investigated, and these systems exhibited absorption patterns similar to those of pristine azulene and distinctive differences from all‐benzenoid analogs. For example, compound **158** possessed three main peaks centered at 326, 375, and 396 nm and a shoulder peak at 345 nm, which were redshifted by 50 nm compared to those of pristine azulene. Furthermore, compounds **158–160** showed narrower bandgaps and retention of solubility and stability under ambient conditions than alternant isomers. More interestingly, the extended azulene‐fused nanographenes **159** and **160** showed fast and reversible responses for the acid‐base redox process, which could be monitored by UV–vis and NMR spectroscopy.

In 2018, Yamada and Aratani reported a naphthalene‐bridged azulene dimer **163** (Scheme 26b).^[^ ^121^ ^]^ They initially attempted to synthesize the naphthalene‐bridged face‐to‐face azulene dimer **164** through twofold Suzuki coupling to investigate through‐space interactions. However, fused‐azulene dimer **163** was found with a yield of 25% in the presence of a typical Suzuki coupling catalysis system instead of **164**. Single‐crystal analysis revealed fusion of the azulene dimer at the 1,1′ positions to form an additional heptagon, which was consistent with the symmetric ^1^H NMR signals. UV–vis–NIR measurements were conducted to investigate the optical property of **163**, indicating an NIR absorption band extended to 900 nm, which was ascribed to the HOMO to LUMO transition. In addition, the aromatic character of **163** was illustrated by theoretical calculations, including NICS and ACID. Naphthalene and two azulene units exhibited aromatic properties, while the central newly formed heptagon showed weak antiaromaticity. The authors proposed that the antiaromaticity of heptagon originated from the 8*π*‐electron cycloheptatrienyl anion.

**Scheme 27:**
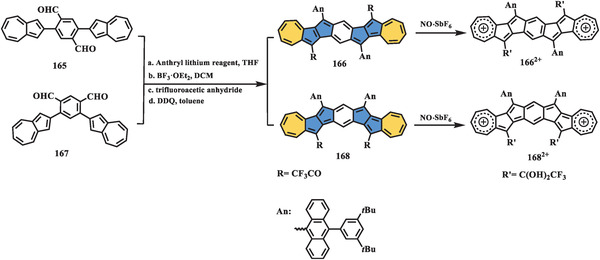
Synthesis of diazuleno‐*s*‐indacene diradicaloids **166** and **168**.^[^ ^122^ ^]^

Additionally, in 2018, Chi et al. presented two azulene‐fused *s*‐indacene isomers, **166** and **168**, with open‐shell character (**Scheme** **27**).^[^ ^122^ ^]^ In this work, dialdehyde precursors **165** and **167** were prepared by two Suzuki couplings. Subsequently, four consecutive steps, namely, nucleophilic addition, F–C alkylation, protection, and oxidation, proceeded toward fully conjugated polyarenes **166** and **168**, in which bulky anthryl groups were used to protect the reactive sites and enhance the solubility, and the trifluoroacetyl groups exhibited acid resistance, allowing for full characterization. X‐ray crystallography revealed that the structures of both compounds **166** and **168** were nearly flat. The bond length analysis and DFT calculations suggested singlet open‐shell character in the ground state mainly arising from the *s*‐indacene subunit, which was also supported by VT‐^1^H NMR, EPR, and SQUID measurements. Furthermore, the NICS value and ACID ring current demonstrated the aromaticity of terminal azulene units and antiaromaticity of the central *s*‐indacene moiety. The electronic behaviors of **166** and **168** were studied by CV. Both compounds showed two reversible low‐lying oxidation waves, at 0.21 and 0.41 V for **166** and 0.1 and 0.39 V for **168** (vs. Fc/Fc^+^), indicating a large tendency toward chemical oxidation. Accordingly, treatment of **166** and **168** with one and two equivalents of NO·SbF_6_ afforded the corresponding radical cation and dicationic species **166** ^2+^ and **168** ^2+^, respectively. From the results of X‐ray crystallographic analysis and DFT calculations, the tropylium rings exhibited aromatic characteristics, whereas the central pentagons were highly antiaromatic and the hexagons were nearly nonaromatic.

## 4 Conclusions and Outlook

In this review, we have summarized the synthetic progress toward azulene‐embedded polyarenes from the 20th century to recent years, not only witnessing advancements in synthetic methodologies and characteristic techniques, but also gaining more fundamental insights into defect‐induced structure–property relationships. Although 5–7 defective pairs can be incorporated into a larger conjugated carbon network, the lack of general cyclization reactions of odd‐membered rings such as pentagons and heptagons limits advances in this renewed field. For example, some of the examples shown in this review were separated through ring‐skeleton rearrangement under Scholl‐type conditions and metal‐mediated alkyne annulation. However, conventional intramolecular F–C reactions also remain powerful in the cyclization process as long as reasonable synthetic strategies are provided.

Despite this progress, difficulties in incorporating joined pentagons and heptagons into different carbon molecular skeletons still restrict the synthesis of defective nanographenes with diverse topologies, thus driving researchers to dedicate more effort to developing efficient and powerful synthetic strategies. Despite the limitations, this field remains dynamic and meaningful to elucidate the appealing physicochemical properties of defective nanographenes, such as optical, electronic, and magnetic behaviors, compared to those of all‐benzenoid analogs, thus providing a fundamental understanding of the defect‐induced changes in molecular geometry and *π*‐electron topology. Accordingly, we look forward to the reaction of more defective nanographenes, such as fragments of pentaheptite and hackelite, and even 1D extended graphene nanoribbons containing different arrangements and orientations of 5–7 pairs may be developed in future works.

## Conflict of Interest

The authors declare no conflict of interest.
